# Balance confidence predicts incident injurious falls in older adults: a longitudinal study

**DOI:** 10.1007/s41999-026-01426-w

**Published:** 2026-02-10

**Authors:** Tewodros Yosef, Julie A. Pasco, Monica C. Tembo, Kara L. Holloway-Kew

**Affiliations:** 1 Deakin University, School of Medicine, IMPACT, The Institute for Mental and Physical Health and Clinical Translation, Geelong, Australia; 2 School of Public Health, College of Medicine and Health Sciences, Mizan-Tepi University, Mizan Teferi, Ethiopia; 3 Department of Medicine – Western Health, The University of Melbourne, St Albans, VIC Australia; 4 Department of Epidemiology and Preventive Medicine, Monash University, Melbourne, VIC Australia

**Keywords:** Injurious falls, Balance confidence, Ageing, Mediation, Youden’s index

## Abstract

**Purpose:**

Falls affect one-third of older adults each year, often resulting in low balance confidence, which is associated with reduced mobility and quality of life. This study examined the association between MFES scores and incident injurious falls and evaluated whether MFES scores mediate the relationship between previous and subsequent falls in older adults.

**Methods:**

Data for participants (n = 952, age ≥ 65 yr) from the Geelong Osteoporosis Study were linked with the Victorian Emergency Minimum Dataset (VEMD). The outcome was the time to first emergency presentation for an injurious fall. Balance confidence was measured using the Modified Falls Efficacy Scale (MFES). Competing risk regression evaluated the MFES score as a predictor of incident injurious falls. Receiver operating characteristic (ROC) analysis determined the optimal MFES cut point for predicting incident injurious falls based on the maximum Youden’s index, while mediation analysis assessed MFES score’s role between prior and subsequent falls.

**Results:**

The median follow-up time was 11.5 years (IQR: 5.9–19.0), with a total of 11,368 person-years. Over the follow-up period, 219 participants (23.0%) experienced at least one incident injurious fall, with an incidence rate of 19.3 per 1000 person-years (95% CI: 16.9–22.0). Low MFES score was a significant predictor of incident injurious falls (aSHR = 1.019, 95% CI: 1.005–1.033). Although an MFES cutoff of 55 was identified, the AUC (0.508) indicated no meaningful discriminative ability. Low MFES score explains 14.9% of the relationship between prior self-reported and subsequent injurious falls.

**Conclusion:**

A low MFES score is associated with incident injurious falls and partially mediates the relationship between previous and subsequent falls. Although an optimal MFES cut point of 55 was identified, its very low specificity and lack of meaningful discriminative ability limit its reliability for prediction. These findings support recommendations, consistent with the World Falls Guidelines, to incorporate assessments of balance confidence—captured through validated measures such as the MFES—into multifactorial fall risk management.

**Supplementary Information:**

The online version contains supplementary material available at 10.1007/s41999-026-01426-w.

## Key summary points

**Aim:**

This study examined the association between MFES scores and injurious falls and evaluated whether MFES scores mediate the relationship between previous and subsequent falls in older adults.

**Findings:**

Each one-point decrease in MFES score was associated with a 1.9% increase in the sub-distribution hazard of injurious falls, and a low MFES score accounted for 14.9% of the relationship between previous and subsequent falls. Although a cutoff of 55 was established, the AUC of 0.508 suggested the measure had little practical predictive value.

**Message:**

Measuring MFES scores is important, as they not only predict incident injurious falls but also partially mediate the link between past and future falls, highlighting their role in guiding targeted fall-prevention strategies.

**Supplementary Information:**

The online version contains supplementary material available at 10.1007/s41999-026-01426-w.

## Introduction

Falls, which affect about one-third of older adults annually [1], are a leading cause of injury-related morbidity and mortality [2] and significantly reduce the quality of life [3]. The *World Falls Guidelines* emphasise this burden—fall-related injuries are common, increasingly so in ageing populations, and contribute substantially to functional decline and healthcare costs [4]. Falls are not only associated with physical injuries but also with psychological and functional consequences, including reduced mobility, loss of confidence, and activity restriction, often referred to as post-fall syndrome [5].

Maintaining balance is fundamental to preventing falls, as impaired postural stability increases the likelihood of losing control during everyday movements [6]. The *World Falls Guidelines* recommend that fall prevention should include a comprehensive assessment of balance and gait, as well as early identification of individuals with diminished balance confidence or fear of falling [4]. Balance confidence, defined as an individual’s cognitive appraisal of their ability to maintain balance, reflects the perceived capacity to perform daily activities safely [7]. Low balance confidence has been linked to activity restriction, deconditioning, and social isolation—all of which heighten vulnerability to falls [8–11].

Empirical evidence on balance confidence as a fall predictor remains mixed. Ellmers et al. [12] found that balance confidence, measured by the Activities-Specific Balance Confidence (ABC) Scale, did not predict future falls; however, other studies have identified balance confidence as a significant protective factor. For instance, Seth et al. [13] reported that a one-point increase in ABC score reduced the odds of falls by 7.3%, while Tsang et al. [14] observed that higher balance confidence was associated with a 43% lower risk of falling. A previous fall is a well-established predictor of subsequent falls, with studies showing that older adults who have fallen are more likely to fall again [15, 16].

Balance confidence is recognised as an important risk factor for falls, as it shapes both psychological and behavioural responses to perceived balance limitations [7, 17]. Following a fall, many older adults begin to view their balance as inadequate, which undermines their confidence in performing daily activities safely [18]. This reduction in balance confidence often leads to activity avoidance [19], resulting in physical deconditioning, muscle weakness [20], and diminished functional capacity [21]—factors that collectively heighten susceptibility to subsequent falls. These interconnected mechanisms underscore the importance of examining psychological and behavioural mediators, such as balance confidence, that may influence the link between previous and subsequent falls.

Balance confidence is commonly evaluated using self-report instruments such as the ABC scale [13, 14, 22, 23], ABC-6 [14, 22], and the Modified Falls Efficacy Scale (MFES) [22], with higher scores reflecting greater confidence in performing daily activities. In this study, we employed the 14-item MFES, an expanded version of the original Falls Efficacy Scale developed by Tinetti et al. [24] and modified by Hill et al. [25], which assesses an individual’s confidence in performing daily activities without falling. To our knowledge, this is the first study to examine balance confidence both as a predictor of injurious falls and as a mediator linking prior and subsequent falls. This study explores balance confidence within the context of global falls prevention guidelines, examines how it influences fall risk, evaluates its role as a mediator between previous and subsequent falls, and identifies the optimal MFES cut point for predicting injurious falls—insights that can guide multifaceted interventions to strengthen confidence, preserve independence, and improve safety in older adults.

## Methods

### Study area and data source

This was a secondary analysis of data from the Geelong Osteoporosis Study [26], a longitudinal, population-based cohort of community-dwelling adults in southeastern Australia. Participants were randomly selected from the electoral roll, with eligibility requiring at least six months of regional residency and the ability to provide informed consent. All participants were community-dwelling and cognitively able to consent at recruitment, though some later transitioned to residential care. A cohort of 1494 women aged 20–94 years was recruited between 1993 and 1997, with follow-up assessment waves conducted at 2, 4, 6-, 8-, 10-, and 15-years post-baseline. Recruitment for men occurred a decade later, between 2001 and 2006, enrolling 1540 participants aged 20–92 years, with follow-up assessments conducted at 5 and 15 years. For this analysis, baseline data were drawn from men’s initial recruitment (2001–2006; n = 1533) and women’s 6-year follow-up (2001–2003; n = 1014). These assessment waves were selected because they provided the most comprehensive data on the primary exposure, balance confidence, as well as other relevant covariates. Among the 2547 participants assessed at these waves, 952 were aged 65 years and older. Since the analysis focused on older adults, only these participants were included, yielding a final sample of 952. The sole exclusion criterion was age below 65 years.

### Study variables

#### Outcome variable

The outcome was the time to first emergency presentation due to an injurious fall. These events were identified through linkage with the Victorian Emergency Minimum Dataset (VEMD), which collects emergency department data from major Victorian public hospitals in Australia, including Warrnambool and District Base Hospital, Hamilton Base Hospital, and University Hospital Geelong [27]. The dataset captured presentations occurring between 1 January 2000 and 31 December 2022. Participants were followed from men’s initial recruitment (2001–2006) and from women’s 6-year follow-up (2001–2003) until the first fall-related emergency presentation, death, or the end of the study period (31 December 2022). Mortality information was obtained via linkage with the National Death Index, maintained by the Australian Institute of Health and Welfare.

#### Exposure variable

The exposure variable, balance confidence, was measured using the MFES [25], which assesses an individual’s confidence in performing daily activities without falling, including tasks such as getting dressed, taking a bath or shower, walking around the house, using public transport, crossing roads, light housekeeping, as well as other everyday activities. The 14-item MFES uses an 11-point scale (0–10) for each activity, where 0 indicates ‘not at all confident’, and 10 indicates ‘completely confident.’ Total scores range from 0 to 140, with lower MFES scores reflecting lower balance confidence. The reliability of the scale was assessed using Cronbach’s alpha, along with item–test correlations (correlation with the total scale including the item), item–rest correlations (correlation with the total scale excluding the item), and the change in alpha if an item was deleted to evaluate each item’s contribution. The scale demonstrated excellent internal consistency (α = 0.953), with all items–rest correlations exceeding 0.70, and removal of any item did not improve reliability.

### Covariates

Weight and height were measured using electronic scales and a Harpenden stadiometer to the nearest 0.1 kg and 0.001 m to calculate body mass index (BMI). Hypertension was defined as systolic pressure ≥ 140 mmHg and/or diastolic pressure ≥ 90 mmHg using an automated device (Takeda Medical UA-751), and the participant was seated. The Timed Up and Go (TUG) test assesses mobility and balance, with times ≥ 12 s indicating slow performance and < 12 s considered normal [28].

Smoking was self-reported, defined as regular use of cigarettes, cigars, or pipes. High alcohol intake was defined as more than 2 standard drinks per day for women and 3 for men, with one standard drink containing 10 g of pure alcohol. Mobility was self-reported using a seven-point scale as previously described [26], with categories of very active, active, sedentary, limited, inactive, chair/bedridden, and bedfast. For analysis, mobility was grouped as high (very active, active) or low (sedentary, limited, inactive, chair/bedridden, and bedfast). Participants' use of mobility assistive devices (e.g., walkers or canes) was recorded (yes/no). A fall was defined as an event in which an individual comes to rest on the ground unintentionally, from a lying, sitting, or standing position [26]. Self-reported previous falls within the previous 12 months were collected at baseline and classified as a binary variable (yes = experienced ≥ 1 fall; no = no falls). Polypharmacy was defined as the use of five or more medications daily [29], including prescription drugs, over-the-counter medications, herbal medicines, and dietary supplements [30]. Stroke and arthritis history were self-reported, while diabetes was diagnosed based on fasting glucose levels ≥ 7.0 mmol/L and/or self-report/medication use.

Pulmonary diseases were defined as the presence of at least one of the following conditions: asthma, emphysema, or chronic bronchitis. Musculoskeletal conditions were assessed via self-report (including osteoarthritis, rheumatoid arthritis, and history of low-trauma fractures at any age, excluding those of the fingers, toes, skull, and face) and measured data (e.g., muscle weakness, low femoral neck bone density (Lunar) with a T-score < − 2.5). Lean soft tissue mass derived from dual-energy X-ray absorptiometry (DXA; Lunar) closely correlates with muscle mass measured by magnetic resonance imaging, and it includes all non-fat, non-bone tissues. For this analysis, leg lean mass—calculated as the combined lean mass of the right and left legs—was used as an indicator of lower-body muscle mass. Hip flexion and abduction forces were assessed using a break test with the Nicholas Manual Muscle Tester (model 01160, Lafayette Instrument Company). Hip flexion and abduction forces were measured three times on each leg without rest between trials, ensuring consistency and accuracy. This testing method is described in detail by Pasco et al. [31]. The maximal force values for hip flexion and abduction forces were converted to Newtons (N) by multiplying by 9.81. To determine hip flexion and abduction muscle strength, these force values were divided by leg lean mass (in kilograms) obtained from DXA scans, resulting in a force-to-mass ratio (N/kg). Cancer data were obtained via data linkage with the Victorian Cancer Registry. Cardiometabolic conditions included stroke, arrhythmias, hypertension, diabetes, obesity (BMI > 30 kg/m^2^), and hypercholesterolemia (total cholesterol ≥ 5.2 mmol/L).

### Statistical analysis

Descriptive statistics were used to summarise participant characteristics and covariates. Means ± SD or medians with interquartile ranges were reported based on variable distribution. Categorical variables were presented as frequencies and percentages. Group differences were assessed using t-tests or Mann–Whitney U tests for continuous variables, and chi-squared tests for categorical variables.

Although Cox regression is the conventional approach for time-to-event analysis, the substantial mortality rate in our dataset (> 10%) constitutes a competing risk for injurious falls, necessitating adjustment to avoid biased estimates [32]. Therefore, we applied competing risk regression (Fine and Gray model) to account for death as a competing event. Cumulative incidence functions were estimated and compared using Gray’s test, and the proportional sub-distribution hazard assumption was evaluated and confirmed to be satisfied. Potential covariates were identified based on theoretical reasoning informed by previous research. Initially, the association between each covariate and the outcome variable was examined using crude (bivariable) analyses to identify candidate variables for the multivariable regression model. Variables with a *p*-value less than 0.25 in the bivariable analysis [33] were considered for inclusion, a threshold that helps retain potentially important predictors while minimising the risk of overfitting [34]. The multivariable model was adjusted for age, sex, alcohol consumption, smoking, polypharmacy, previous self-reported falls, mobility, use of mobility assistive devices, TUG test, and hip flexion and abduction strength. An adjusted sub-distribution hazard ratio (aSHR) with 95% confidence interval was reported, with statistical significance set at *p* < 0.05. No significant interactions were detected, and multicollinearity was not a concern, with all VIF values below 2.

Receiver operating characteristic (ROC) curve analysis, including sensitivity, specificity, and AUC, was used to assess the predictive ability of balance confidence for incident injurious falls. The optimal cut point was determined using Youden’s index [35]. A mediation analysis was performed with previous self-reported falls as the exposure variable (X), MFES score as the mediator (M), and injurious falls as the outcome variable (Y). The mediation effect was evaluated using the bootstrapping method with 5000 resampling iterations. The direct effect (DE) measures the effect of previous self-reported falls without mediation or interaction, while the indirect effect (IE) reflects the effect solely due to mediation. The total effect is the sum of DE and IE [36]. The proportion mediated was calculated using the formula [(indirect effect) / (total effect) *100] [36]. The data were analysed using Stata version 18 (Stata Corp. 2023. Stata Statistical Software: Release 18. College Station, TX: Stata Corp LLC).

## Results

### Participant characteristics by incident injurious falls status

Among men, fallers were older than non-fallers and had a lower mean BMI. Among women, fallers were also older than non-fallers and took longer to complete the TUG test (Table 1). During the follow-up period, 431 (45.3%) participants died, and 219 (23.0%) experienced falls.

**Table 1:** Sex-stratified demographics, body composition, and function by incident injurious falls status. Data are presented as median (IQR) or mean ± SD

Variables	Men			Women		
	Non-fallers	Fallers	*p*-value	Non-fallers	Fallers	*p*-value
Age (yr)	76 (71–82)	79 (73–84)	0.001	75 (69–80)	78 (72–83)	0.009
Height, m	1.75 ± 0.05	1.74 ± 0.05	0.032	1.57 ± 0.07	1.57 ± 0.06	0.620
Body mass, kg	85.9 ± 9.0	84.2 ± 10.0	0.065	67.7 ± 13.0	67.1 ± 14.1	0.711
Body mass index, kg/m^2^	28.1 ± 1.38	27.8 ± 1.62	0.021	27.5 ± 5.11	27.3 ± 5.14	0.776
Leg lean mass, kg	18.4 ± 3.13	18.5 ± 2.97	0.695	11.0 ± 2.35	11.2 ± 2.03	0.444
Relative leg lean mass, kg/m^2^	2.99 ± 0.52	3.05 ± 0.49	0.319	2.42 ± 0.43	2.34 ± 0.50	0.187
TUG test (second)	9.5 ± 2.9	9.4 ± 2.3	0.643	8.7 ± 3.0	9.8 ± 3.0	0.003

*IQR* Interquartile range; *SD* Standard deviation

### Distribution of variables by survival status

More participants with cardiometabolic conditions were in the censored group (64.3%) compared to the deceased (36.9%) and event (46.3%) groups (*p* < 0.001). Previous fall history was more common in the event group (41.0%) compared to the censored (23.2%) and deceased (37.4%) groups (*p* < 0.001) (Table 2).

**Table 2:** Description of baseline characteristics across survival status categories. Data are presented as n (%)

Variables	Survival status			X^2^ (*df* = 2)	*p-*value
	Censored (n = 302)	Deceased (n = 431)	Event (n = 219)		
Sex (female)	164 (54.3)	104 (24.1)	94 (42.9)	71.50	< 0.001
Cardiometabolic^a^ (yes)	191 (64.3)	158 (36.9)	101 (46.3)	52.96	< 0.001
Pulmonary^b^ (yes)	52 (17.4)	73 (17.1)	36 (16.5)	0.07	0.966
Cancer (yes)	71 (23.7)	75 (17.5)	48 (22.0)	4.48	0.106
Musculoskeletal^c^ (yes)	111 (37.0)	127 (29.7)	66 (30.4)	4.66	0.097
Polypharmacy^d^ (yes)	22 (7.3)	74 (17.3)	25 (11.5)	16.1	< 0.001
High alcohol intake (yes)	126 (43.3)	143 (35.8)	69 (32.2)	7.22	0.027
Smoking (yes)	27 (11.1)	30 (9.1)	19 (10.2)	0.64	0.726
Poor mobility (yes)	58 (19.3)	139 (32.6)	64 (29.4)	15.97	< 0.001
Walking aid use (yes)	13 (4.3)	47 (11.0)	20 (9.2)	10.32	0.006
TUG test (slow)	8 (2.8)	96 (25.1)	34 (16.8)	62.34	< 0.001
Fall history (yes)	70 (23.2)	160 (37.4)	89 (41.0)	22.76	< 0.001

^a^Cardiometabolic conditions included hypertension, stroke, arrhythmias, diabetes, obesity (BMI > 30 kg/m^2^), and hypercholesterolemia (total cholesterol ≥ 5.2 mmol/L)

^b^pulmonary disease included asthma, emphysema, and chronic bronchitis

^c^Musculoskeletal conditions included muscle weakness, osteoarthritis, rheumatoid arthritis, low femoral neck bone mineral density, and previous low-trauma fractures (excluding fractures of the fingers, toes, skull, and face)

^d^Polypharmacy (defined as ≥ 5 medicines)

*n* frequency; *df* degree of freedom

### Incidence of injurious falls

The median follow-up time was 11.5 years (IQR: 5.9–19.0), with a total of 11,368 person-years. The median time to the first incident of injurious falls was 8.0 years (IQR: 4.0–12.2). Over the follow-up period, 219 participants (23.0%) experienced at least one incident injurious fall, with an incidence rate of 19.3 per 1000 person-years (95% CI: 16.9–22.0). Analysis of cumulative incidence curves reveals a consistent rise in fall risk across selected variables—sex, TUG, polypharmacy, and history of falls (Supplementary Fig. 1).

### Modified fall efficacy scale score by incident injurious falls status

Fallers and non-fallers exhibited significantly different confidence levels across most activities on the MFES. The largest differences were observed in tasks such as taking a bath or shower (mean difference = 0.51, 95% CI: 0.26, 0.77, *p* < 0.001), getting in/out of bed (0.29, 95% CI: 0.06, 0.51, *p* = 0.012), walking inside the house (0.29, 95% CI: 0.12, 0.46, *p* = 0.001), and reaching into cabinets or closets (0.61, 95% CI: 0.36, 0.85, *p* < 0.001) (Table 3). The overall MFES score of the participants was highly skewed and concentrated toward the maximum, with a mean of 132.2 (SD ± 18.6) and a median of 140 (IQR: 134–140).

**Table 3:** Differences in MFES scores between incident injurious fallers and non-fallers

Modified fall efficacy scale items		Difference in score	95% CI	*t*	*df*	*p*-value
1	Get dressed and undressed	0.07	[− 0.17, 0.31]	0.57	943	0.571
2	Prepare a simple meal	0.06	[− 0.15, 0.27]	0.55	942	0.582
3	Take a bath or a shower	0.51	[0.26, 0.77]	3.93	942	< 0.001
4	Get in/out of a chair	0.23	[− 0.00, 0.46]	1.93	942	0.054
5	Get in/out of bed	0.29	[0.06, 0.51]	2.52	942	0.012
6	Answer the door or telephone	0.18	[0.00, 0.36]	2.01	942	0.044
7	Walk around the inside of your house	0.29	[0.12, 0.46]	3.29	942	0.001
8	Reach into cabinets or closet	0.61	[0.36, 0.85]	4.92	943	< 0.001
9	Light housekeeping	0.21	[− 0.02, 0.44]	1.8	942	0.072
10	Simple shopping	0.26	[− 0.01, 0.53]	1.9	941	0.057
11	Using public transport	0.41	[0.08, 0.73]	2.44	938	0.015
12	Crossing roads	0.21	[− 0.09, 0.51]	1.38	942	0.168
13	Light gardening or doing the laundry	0.04	[− 0.25, 0.33]	0.26	941	0.793
14	Using front or rear steps at home	0.21	[− 0.07, 0.48]	1.47	941	0.140

### MFES score differences

Participants with musculoskeletal conditions had significantly lower MFES scores (mean difference = − 2.76, *p* = 0.034) compared with those without such conditions. Similarly, participants with high alcohol intake had lower MFES scores (mean difference = − 2.88, *p* = 0.017) than those with low or no intake. Participants with mobility limitations scored significantly lower on the MFES (mean difference = − 4.06, *p* = 0.003) compared with those without mobility limitations. Participants who used mobility assistive devices had markedly lower MFES scores (mean difference = − 8.10, *p* < 0.001) than those who did not use such devices. Participants with a history of falls scored significantly lower on the MFES (mean difference = − 4.94, *p* < 0.001) compared with those with no previous falls (Table 4).

**Table 4:** An independent t-test results for MFES score differences by demographic, health, and behavioural factors

Variables	Difference in score	95% CI	t-statistic	*df*	*p*-value
Sex (male vs female)	− 1.05	[− 3.49, 1.39]	− 0.85	943	0.397
Cardiometabolic (no vs yes)	− 2.22	[− 4.60, 0.16]	− 1.83	934	0.068
Pulmonary (no vs yes)	− 1.15	[− 4.31, 2.00]	− 0.72	936	0.474
Cancer (no vs yes)	2.50	[− 0.44, 5.45]	1.67	937	0.096
Musculoskeletal (no vs yes)	− 2.76	[− 5.30, − 0.22]	− 2.13	935	0.034
Polypharmacy (no vs yes)	0.60	[− 2.98, 4.17]	0.33	937	0.744
High alcohol intake (no vs yes)	− 2.88	[− 5.24, − 0.52]	− 2.40	899	0.017
Smoking (no vs yes)	0.41	[− 3.85, 4.68]	0.19	755	0.850
Mobility (active vs inactive)	4.06	[1.41, 6.71]	3.01	935	0.003
Walking aid use (no vs yes)	8.10	[3.86, 12.33]	3.75	935	< 0.001
TUG test (normal vs slow)	14.16	[11.81, 16.51]	11.82	871	< 0.001
Fall history (no vs yes)	4.84	[2.37, 7.30]	3.85	940	< 0.001

### Modified fall efficacy scale cut point to predict incident injurious falls

The MFES cut point of 55 was identified as optimal for predicting incident injurious falls (99.1% sensitivity, 1.3% specificity). The area under the receiver operating characteristic curve (AUROC) for the MFES cut point predicting incident injurious falls was 0.508. After dichotomizing the MFES score based on the optimal cut-point to predict incident injurious falls, binary logistic regression analysis revealed that participants with an MFES score < 55 had 39% increased odds of injurious falls (odds ratio = 1.39, 95% CI: 1.06–3.55). Although some fallers had high MFES scores and vice versa, non-fallers had a higher mean score (133.0 ± 18.1) than fallers (129.7 ± 19.9; *p* = 0.021).

### MFES as a predictor of incident injurious falls

In the multivariable model, the adjusted sub-distribution hazard ratio (aSHR) for MFES score was 1.019 (95% CI: 1.005–1.033), with *p* = 0.007. This means that each one-point decrease in MFES score was associated with a 1.9% increase in the sub-distribution hazard of injurious falls (Table 5). Low MFES score explained 14.9% of the relationship between previous self-reported falls and subsequent injurious falls.

**Table 5:** A competing risk regression analysis of MFES score as a predictor of incident injurious falls

Variable	Rate of incident injurious falls			
	cSHR (95%CI)	*p*-value	aSHR (95%CI)	*p*-value
MFES score	1.007 (1.002–1.013)	**0.009**	1.019 (1.005–1.033)	**0.007**

*aSHR* adjusted sub-distribution hazard ratio; *CI* confidence interval; *cSHR* crude sub-distribution hazard ratio; *MFES* modified fall efficacy scale

## Discussion

This longitudinal study examined the association between MFES scores and injurious falls and evaluated whether MFES scores mediate the relationship between previous and subsequent falls in older adults. In this cohort, the incidence rate of injurious falls was 19.3 per 1000 person-years (95% CI: 16.9–22.0). The MFES score was a significant predictor: each one-unit decrease in MFES score increased the rate of injurious falls by 1.9%. The MFES score also accounted for 14.9% of the association between previous self-reported falls and subsequent injurious falls. The optimal MFES cut point for predicting incident injurious falls was 55.

During follow-up, 23.0% of participants experienced at least one fall, with an incidence of 19.3 per 1000 person-years (95% CI: 16.9–22.0). This incidence was higher than that reported by Kenis et al. (13.2% over two years) [37] and Sasidharan et al. (20.1% over one year; 31 per 100 person-years) [38], but lower than the rates observed by O’Loughlin et al. (29% over four years; 496.8 per 1000 person-years) [39] and Lavedán et al. (35.2% over two years in adults ≥ 75 years) [40]. These variations are likely due to differences in definitions of fall and measurement methods.

The relatively lower incidence rate in this study may also reflect differences in participant age and follow-up duration. While Lavedán et al. [40], specifically focused on older adults aged ≥ 75 years, our study included a broader age range, encompassing individuals aged ≥ 65 years. Fall risk increases substantially with age [41], influenced by factors such as chronic conditions (e.g., osteoporosis, arthritis), cognitive impairments, vision problems, medication effects, muscle weakness, and balance issues [42–45]. Adults aged ≥ 75 years typically demonstrate higher fall rates, partly due to age-related declines in muscle strength of 3.6–5.0% per year from age 65 onward [46].

In contrast to previous studies [38–40], which typically had shorter follow-up periods, this study benefited from a long-term follow-up with a median duration of 11.5 years. Follow-up duration directly affects incidence rate calculations because person-time increases as participants remain in the cohort [47]. A longer follow-up can lower incidence rates when events do not increase proportionally. Additionally, we captured only hospital-presenting falls, which may reflect more severe events, as individuals with minor falls may not seek emergency care.

The MFES score was strongly linked to fall risk. This study explored the relationship between MFES score and injurious fall incidence, indicating that each one-unit reduction in MFES score was associated with a 1.9% increase in the rate of injurious falls. This suggests that individuals with higher MFES scores—indicating greater confidence performing daily activities without falling—may have a lower risk because they remain active and maintain better physical function. This aligns with previous studies showing that MFES scores predict future falls [37, 48], as low MFES scores are associated with reduced physical activity [19], muscle weakness, impaired balance [20, 21], and a heightened risk of falling [10]. MFES scores were highly skewed and clustered near the maximum, reflecting a pronounced ceiling effect and limited variability in participants’ MFES scores. As a result, the reported effect per unit increase should be interpreted cautiously, since small differences on the scale may have minimal practical significance. These findings suggest that, while statistically significant, the effect estimates may have limited clinical relevance and should be understood in the context of the restricted score distribution.

Although the MFES cut point of 55 yielded extremely high sensitivity (99.1%), its specificity was very low (1.3%). This indicates that nearly all participants who did not experience an injurious fall would be incorrectly classified as at risk. The AUROC of 0.508 further demonstrates poor discriminative ability—very close to chance—showing that the MFES score, when used alone, is not an effective stand-alone predictor of injurious falls. While the dichotomised MFES score remained statistically associated with injurious falls (OR = 1.39, 95% CI: 1.06–3.55), this reflects only a modest risk increase and does not support meaningful clinical utility.

A cautious interpretation is warranted because MFES was measured several years before some fall events occurred. During this time, changes in confidence, physical function, health status, or environmental exposures could reduce predictive accuracy. The extremely low specificity also raises concerns about false positives, potentially causing unnecessary worry among older adults without providing accurate or actionable risk stratification. Overall, while the MFES score remains an important psychosocial correlate of fall risk, the identified cut-point has limited practical value for screening or stratifying individual fall risk. MFES should be used as part of a broader, multifactorial assessment rather than a stand-alone predictive tool.

A difference is considered clinically important only when it reflects a meaningful change in real-world function, not merely a statistically significant finding [49]. Because no minimal clinically important difference (MCID) has been established for the MFES, the practical relevance of the observed 3.3-point difference between fallers and non-fallers is uncertain. To aid interpretation, we calculated an effect size: the Cohen’s d for this difference was 0.17, which falls below the threshold for a small effect (d = 0.20) [50]. Given the wide scoring range of the MFES (0–140) and this minimal effect size, the statistically significant difference observed here is unlikely to represent a clinically important change in the modified fall efficacy scale score.

Mediation analysis assumes that the exposure precedes the mediator, which in turn precedes the outcome [51]. In this study, previous self-reported falls and MFES score were measured concurrently, limiting the ability to establish temporality between these variables. However, a clear temporal relationship existed between the MFES score and subsequent injurious falls. Mediation remains plausible when supported by prior evidence of temporal ordering: previous falls can reduce confidence in performing daily activities, leading to diminished activity, reduced physical capacity, and ultimately higher risk of subsequent injurious falls [52, 53]. Consistent with this pathway, the present study found that a history of falls was linked to lower MFES scores, with approximately 14.9% of the effect of previous falls on subsequent falls mediated through MFES, suggesting that part of the impact of prior falls operates by reducing MFES scores. These findings highlight low MFES score as a modifiable psychological factor in the falls pathway and underscore the importance of interventions aimed at enhancing confidence to disrupt the cycle of recurrent falls.

A low MFES score may serve as a mediator by influencing both psychological and behavioural pathways. Experiencing a fall can reduce confidence and increase fear of falling [5, 52, 54], prompting voluntary activity restriction [55]. Although this coping strategy may seem protective in the short term [53], it can lead to physical deconditioning, muscle weakness, and reduced functional capacity [20], ultimately raising the risk of future falls [10]. By affecting both behaviour and physical function, the MFES score plays a central role in the fall pathway. These findings emphasise the importance of interventions that address both physical and psychological determinants of mobility to reduce injurious falls in older adults.

### Strengths and limitations

This study benefits from a population-derived sample, long-term follow-up, and robust analytic methods, including competing-risk regression to account for deaths. MFES was used as a validated measure of balance confidence. Linked medical records reduced self-report bias but captured only injurious falls, potentially missing events among individuals who did not seek care due to socio-cultural or mobility barriers. The low specificity of the MFES cut-point may lead to false-positive risk classification. Although incident injurious falls were measured prospectively, previous self-reported falls and MFES score were assessed concurrently, limiting causal mediation inference. Therefore, the mediation findings should be interpreted as associative rather than causal.

## Conclusion

A low MFES score is a significant predictor of incident injurious falls and partially mediates the relationship between previous and subsequent falls, accounting for 14.9% of this association. Although an optimal MFES cut point of 55 was identified, its low specificity and lack of meaningful discriminative ability limit its reliability for risk stratification. These findings support recommendations, consistent with the World Falls Guidelines, to incorporate assessments of balance confidence—captured through validated measures such as the MFES—into multifactorial fall risk management. Overall, the results highlight the role of MFES as a supportive tool for guiding targeted interventions aimed at maintaining functional independence and reducing fall-related adverse outcomes.

## Supplementary Information

Below is the link to the electronic supplementary material.

Supplementary file1 (DOCX 229 KB)

## Data Availability

The data that support the findings of this study are available from the Victorian Minimum Emergency Dataset and the Geelong Osteoporosis Study. Restrictions apply to the availability of these data, which were used under approvals for this study. Data are available from the authors with the permission of the Victorian Minimum Emergency Dataset.

## Abbreviations

BMI: Body mass index
MFES: Modified fall efficacy scale
SHR: Sub-distribution hazard ratio
TUG test: Timed up and go test
VEMD: Victorian emergency minimum dataset
