# Supplementary material for: Balance confidence predicts incident injurious falls in older adults: a longitudinal study
Source: Eur Geriatr Med. 2026 Feb 10;17(2):771–80. doi: 10.1007/s41999-026-01426-w (PMC13109150; doi:10.1007/s41999-026-01426-w)
Supplement: Supplementary file 1 — Supplementary file1 (DOCX 229 KB) [file 41999_2026_1426_MOESM1_ESM.docx]

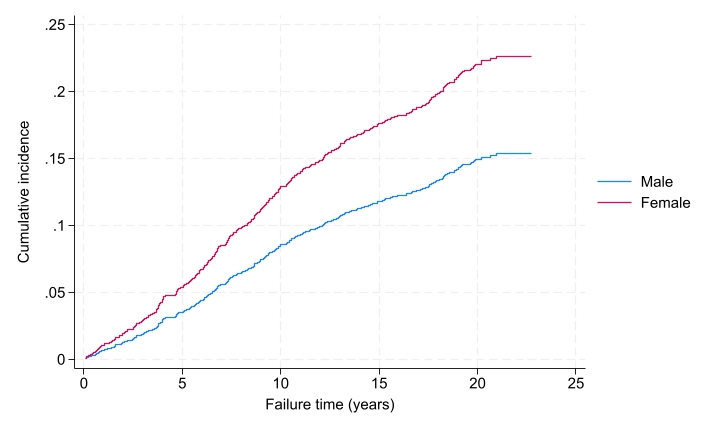


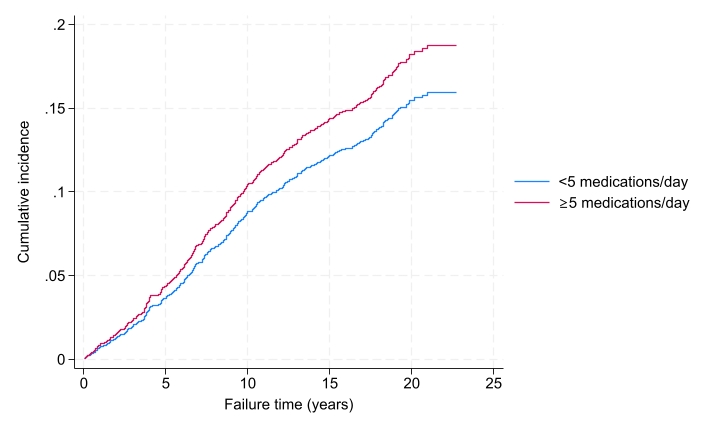


1. c)


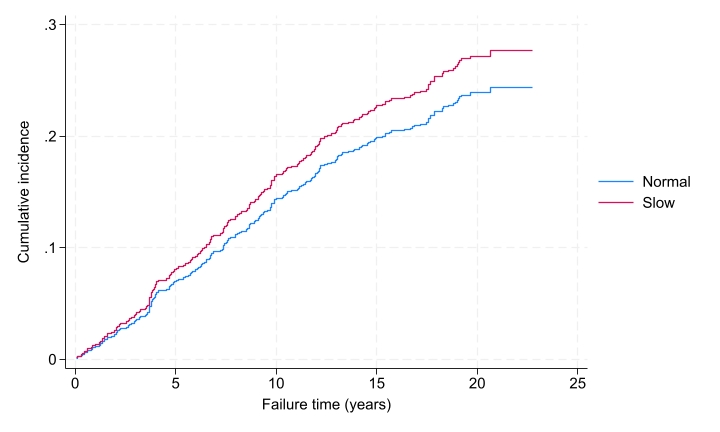


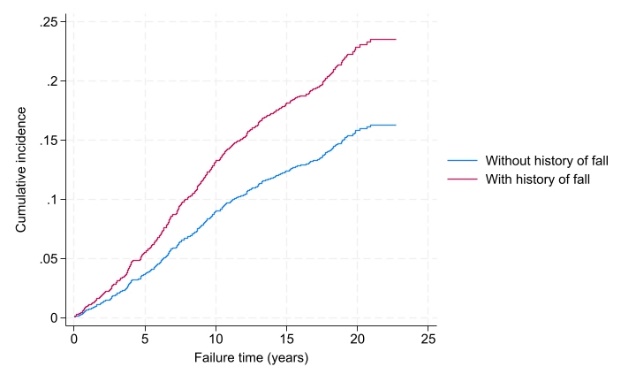


b) d)

Supplementary figure 1: The cumulative incidence function curves for incident injurious falls by (a) sex, (b) Time Up and Go test, (c) polypharmacy, and (d) history of falls, considering death as a competing event.
